# Congruence between cytochrome oxidase I (COI) and morphological data in *Anuraphis* spp. (Hemiptera, Aphididae) with a comparison between the utility of the 5’ barcode and 3’ COI regions

**DOI:** 10.3897/zookeys.529.6081

**Published:** 2015-10-26

**Authors:** Giuseppe E. Massimino Cocuzza, Silvia Di Silvestro, Rosanna Giordano, Carmelo Rapisarda

**Affiliations:** 1 Dipartimento di Agricoltura, Alimentazione e Ambiente, Università di Catania, via S. Sofia 100, 95123 Catania, Italy; 2 Centro di Ricerca per l’Agrumicoltura e le Colture Mediterranee, Corso Savoia 190, 95024 Acireale, Italy; 3 Department of Biology, University of Puerto Rico, San Juan, PR 00931, USA

**Keywords:** Insects, aphids, taxonomy, species identification

## Abstract

The discrimination of species in the genus *Anuraphis* is particularly difficult due to the overlap of morphological characters. In this study, we used the 5’ (barcode) and 3’ regions of cytochrome oxidase I (COI) to test their utility in the identification of species in this genus as well as closely related species. Both regions were useful to discriminate all the species tested. However the non-barcode 3’ region resulted in higher resolution and support for species relationships when the data were analyzed using both Maximum Likelihood and MrBayes. We propose the development of an integrated database that encompasses morphological, molecular, life-cycle, host plant and bibliographic information to facilitate and increase the accuracy of aphid identification.

cytochrome oxidase I

## Introduction

Aphids are sap-sucking insects. Currently there are 5012 valid species (Favret 2014) associated with plants belonging to various botanical groups. Many species have a heteroecious life cycle that includes alternating between a primary host plant (usually a tree) and a secondary host (usually an herbaceous species). The genus *Anuraphis* Del Guercio presently ascribed to the tribe Macrosiphini includes a small number of taxonomically well-defined species, *Anuraphis subterranea* (Walker, 1852), *Anuraphis farfarae* (Koch, 1854), *Anurahis catonii* Hille Ris Lambers, 1935, *Anuraphis pyrilaseri* Shaposhnikov, 1950, *Anuraphis cachryos* Barbagallo & Stroyan, 1982, *Anuraphis ferulae* Shaposhnikov, 1995 and *Anuraphis shaposhnikovi* Barbagallo & Cocuzza, 2003. In addition, Remaudière and Remaudière (1997) reported four other nominal species (i.e., *Anuraphis capparidis* Nevsky, 1929, *Anuraphis cortusae* Nevsky, 1929, *Anuraphis floris* Monzen, 1934 and *Anuraphis katsurae* Shinji, 1952). However, the generic placement of *Anuraphis capparidis* has been questioned by Blackman and Eastop (2006) who noted that, based on the original description, this is probably not an *Anuraphis* species but an immature *Aphis* sp. The recognized *Anuraphis* species are distributed in the Ponto-Mediterranean area of the western Palaearctic region. A common trait of almost all *Anuraphis* species is the use of Apiaceae as host plants, with the exception of *Anuraphis farfarae* that feeds on Asteraceae (*Tussilago*, *Petasites* and *Hieracium*). Some populations of *Anuraphis subterranea*, *Anuraphis pyrilaseri*, *Anuraphis farfarae* and *Anurahis catonii* have been shown to be heteroecious holocyclic with *Pyrus* spp. (Rosaceae) as primary host plants (Shaposhnikov 1951; Kolesova 1972; Lampel and Meyer 2007). However, some populations of *Anuraphis farfarae* (Shaposhnikov & Sharov, 1978), and probably other species, are solely anholocyclic on secondary host plants. For *Anuraphis cachryos*, *Anuraphis shaposhnikovi* and *Anuraphis ferulae* the primary host plants remain to be determined.

*Anuraphis farfarae* (pear-colt’s foot aphid) and *Anuraphis subterranea* (pear-hogweed aphid) have been reported in the literature as pests of pear, where they cause direct damage to young foliage in spring (Kolesova 1972). However, damage due to their infestation has a negligible effect on production (Alford 2014).

All species belonging to the genus *Anuraphis* are morphologically similar to each other but easily discriminated from other genera. The main morphological features of the genus are an almost flat frontal profile, as a result of the minimally developed antennal tubercles, and a short cauda. Moreover, *Anuraphis* shares with a few other genera of Macrosiphini a typical spinulose ornamentation of siphunculi and a well-developed, often almost complete set of dorsal tubercles (both marginal and spinal). However, as already reported for other groups of aphids, the morphometric similarity among *Anuraphis* species leads to an overlap that renders their discrimination to species level difficult (Stroyan 1984; Heie 1986). Barbagallo and Cocuzza (2003) published a morphological key to discriminate viviparous morphs (for both apterae and alate) of *Anuraphis* species and a discriminant function to separate *Anuraphis subterranea* and *Anuraphis shaposhnikovi*. However, the discrimination of *Anuraphis subterranea* and *Anuraphis shaposhnikovi* using only morphological characters requires the skills of an experienced researcher, especially when specimens are collected on primary host plants or when the secondary host is unknown.

In some genus (e.g. *Aphis*), a recurrent and difficult problem in using only morphological characters to identify aphids is that for many species there are insufficient diagnostic characters, resulting in their identification being partially based on host plant association and life cycle characteristics (Stroyan 1984; Heie 1986). However, due to incomplete and/or missing knowledge of many aphid/plant associations, the use of this criterion to identify aphid species, could lead to misidentification (Stroyan 1984; Coeur d’acier et al. 2007). Many studies have used the 5’ region of the cytochrome oxidase I gene (COI), more commonly referred to as the DNA barcode region, as a useful tool to discriminate various groups of insects (Hebert et al. 2003a, b, Deng et al. 2012; Derocles et al. 2012; Williams et al. 2012; Julsirikul et al. 2013), including aphid species (Coeur d’acier et al. 2008; Foottit et al. 2008, 2009a, b, c; Miller and Foottit 2009; Wang and Qiao 2009; Kim et al. 2010; Lee et al. 2011; Zhang et al. 2010, 2011; Wang et al. 2011; Chen et al. 2013; Massimino Cocuzza and Cavalieri 2014). However, especially in some insect groups such as Aphididae, the DNA barcode region, due to low genetic diversity at this marker, was no more informative than morphological characters (Foottit et al. 2008; Lee et al. 2011). For instance, results obtained using the COI barcode region with adelgids were inadequate for the purpose of discriminating species that were morphologically indistinguishable or belonged to a species-complex (Žuroková 2010). Other studies have shown that the COI barcode region discriminated 96% of aphid taxa tested (Foottit et al. 2008).

Ideally the description of a species should result from a synthesis of information that encompasses morphological, molecular, biological, biogeographical, physiological, ecological and bibliographical data (Dayrat 2005; De Salle 2006; Waugh 2007; Padial et al. 2010; Taylor and Harris 2012), however, this compendium of information is lacking for the great majority of species.

This study was undertaken to improve the current taxonomic knowledge of the various taxa belonging to the genus *Anuraphis* by testing the utility of the COI gene, specifically comparing the widely used barcode 5’ region with the much less studied 3’ region, as a molecular tool for their identification. A further goal is to compare the results obtained with the COI gene to those previously published using only morphological characters (Barbagallo and Cocuzza 2003).

## Materials and methods

This study was conducted with seven species (Table 1) belonging to the genus *Anuraphis*. Unfortunately, it was not possible to include *Anuraphis ferulae*, a species recorded only from Tajikistan on *Ferula* sp. When possible, species were collected in different geographic locations and on different host plants. Taxonomic nomenclature follows Remaudière and Remaudière (1997). Two samples of *Nearctaphis bakeri* (Cowen, 1895) were included in the analysis. The genus *Nearctaphis* is considered the vicariant (or sister) Nearctic relative of *Anuraphis*, from which it differs morphologically due to the lack of spinal tubercles, and biologically by the use of *Malus* sp. as a primary host plant and Fabaceae and Scrophulariaceae as secondary hosts (Hille Ris Lambers 1970). In addition, samples of *Roepkea marchali* Hille Ris Lambers, *Brachycaudus jacobi* Stroyan and *Aphis fabae* Scopoli, were used as out-groups. Collections of aphid colonies were made on individual plants and at least two individuals were sequenced per collection. Details regarding the specimens used in this study (host plants, collection locality, sampling date and gene bank accession numbers) can be found in Table 1. For each sample, 5–6 apterae and alate individuals were slide-mounted for morphological identification. Specimens were morphologically identified by S. Barbagallo using characters in the keys provided by Heie (1992), Barbagallo and Cocuzza (2003) and Blackman (2010). Specimen slides are stored in the Aphididae collection of S. Barbagallo (Department of Agriculture, Food and Environment, University of Catania).

**Table 1.** Summary of information on samples used in the molecular analysis.

Voucher code	Species	Host plant	Location	Sampling date	GeneBank accession N°
S03189	*Anuraphis farfarae* Koch	*Tussilago farfara*	40,0970N/15,8131E Lauria (Potenza, Basilicata)	25 Jun. 03	KT878791
S03190	*Anuraphis farfarae*	*Tussilago farfara*	39,8762N/16,0050E Mormanno (Cosenza, Calabria)	25 Jun. 03	KT878792
S13572	*Anuraphis farfarae*	*Tussilago farfara*	46,5606N/12,1285E Cortina d’Ampezzo (Bolzano, Trentino Alto Adige)	18 Sep. 13	KT878793
S03157	*Anuraphis pyrilaseri* Shaposhnikov	*Magydaris pastinacea*	37,9795N/12,7637E Buseto Palizzolo (Trapani, Sicily)	6 Jun. 03	KT878794
S03171	*Anuraphis pyrilaseri*	*Thapsia garganica*	37,9258N/15,7602E Rognudi (Reggio Calabria, Calabria)	9 Jun. 03	KT878795
S03141	*Anuraphis pyrilaseri*	*Ferula communis*	37,6345N/15,0744E Trecastagni (Catania, Sicily)	15 May 03	KT878797
S03146	*Anuraphis pyrilaseri*	*Ferula communis*	38,0229N/15,3890E Fiumedinisi (Messina, Sicily)	17 May 03	KT878799
S03152	*Anuraphis pyrilaseri*	*Thapsia garganica*	37,8152N/15,1869E Piedimonte Etneo (Catania, Sicily)	28 May 03	KT878796
S03147 CBGP#ACOE2024 GBMIN37806 CBGP#ACOE2050 CBGP#ACOE1998	*Anuraphis pyrilaseri* *Anuraphis pyrilaseri* *Anuraphis pyrilaseri* *Anuraphis pyrilaseri* *Anuraphis pyrilaseri*	*Ferula communis* Not reported Not reported Not reported Not reported	38,0440N/15,4309E Itala (Messina, Sicily) 37,7863N/15,2337E Fiumefreddo (Catania, Sicily) Not reported 37,7826N/15,1325E Sant’Alfio (Catania, Sicily) 37,7827N/15,1418E Linguaglossa (Catania, Sicily)	17 May 03 27 May 06 Not reported 30 May 06 23 May 06	KP714117 ACEA860 GU568501 ACEA880 ACEA839
S03144	*Anurahis catonii* HRL	*Pimpinella major*	38,0505N/15,4343E Itala (Messina, Sicily)	17 May 03	KT878815
S03173	*Anurahis catonii*	*Pimpinella peregrina*	37,9937N/15,9250E Bova (Reggio Calabria, Calabria)	9 Jun. 03	KT878816
S12477	*Anurahis catonii*	*Pimpinella peregrina*	37,1334N/15,0165E Sortino (Siracusa, Sicily)	25 May 12	KT878817
S03179	*Anuraphis cachryos* Barb. & Str.	*Cachrys sicula*	37,3619N/15,0219E Scordia (Catania, Sicily)	15 Jun. 12	KT878818
S03180	*Anuraphis cachryos*	*Cachrys sicula*	36,7765N/14,5989E Donnalucata (Ragusa, Sicily)	15 Jun. 12	KT878819
S12423	*Anuraphis cachryos*	*Cachrys sicula*	36,7766N/14,5990E Donnalucata (Ragusa, Sicily)	2 May 12	KT878820
S14599 CPGP#ACOE1057	*Anuraphis cachryos* *Anuraphis cachryos*	*Cachrys libanotis* Not reported	37,3080N/14,8587E Lentini (Siracusa, Sicily) 42,7869N/3,0361 Languedoc-Roussillon (France)	13 Jun. 13 30 Oct. 00	KT878821 ACEA353
S03181	*Anuraphis subterranea* (Walker)	*Heracleum pyrenaicum*	37,9756N/14,9516E Floresta (Messina, Sicily)	22 Jun. 03	KT878800
S03182	*Anuraphis subterranea*	*Heracleum pyrenaicum*	37,9808N/15,1435E Novara di Sicilia (Messina, Sicily)	22 Jun. 03	KT878801
S12517	*Anuraphis subterranea*	*Heracleum sphondylium*	37,9020N/13,9999E Isnello (Palermo, Sicily)	3 Jul. 12	KT878804
S03191	*Anuraphis subterranea*	*Pastinaca sativa*	39,8761N/16,0038E Mormanno (Cosenza, Sicily)	25 Jun. 03	KT878805
S03163	*Anuraphis subterranea*	*Heracleum pyrenaicum*	37,8801N/14,0283E Petralia Sottana (Palermo, Sicily)	6 Jun. 03	KT878802
S03184 CBGP#ACOE2053 CBGP#ACOE2060 CBGP#ACOE645 CBGP#ACOE1068	*Anuraphis subterranea* *Anuraphis subterranea* *Anuraphis subterranea* *Anuraphis subterranea* *Anuraphis subterranea*	*Heracleum pyrenaicum* Not reported Not reported Not reported Not reported	37,9756N/14,9516E Floresta (Messina, Sicily) 37,9216N/14,957E Randazzo (Catania, Sicily) 37,9921N/14,9306E Floresta (Messina, Sicily) 44,8893N/1,4062E Peryllac-et-Millac (France) 42,8742N/2,1829E Quillan (France)	22 Jun. 03 30 May 06 30 May 06 2 Jun. 99 21 May 01	KT878803 ACEA883 ACEA890 ACEA164 ACEA367
S03160	*Anuraphis shaposhnikovi* Barb. & Coc.	*Magydaris pastinacea*	37,9795N/12,7637E Buseto Palizzolo (Trapani, Sicily)	6 Jun. 03	KT878808
S03143	*Anuraphis shaposhnikovi*	*Opopanax chironium*	37,9075N/15,1211E Francavilla di Sicilia (Messina, Sicily)	16 May 03	KT878809
S03166	*Anuraphis shaposhnikovi*	*Opopanax chironium*	37,9917N/15,9309E Bova Sup. (Reggio Cal., Calabria)	9 Jun. 03	KT878810
S14589 CBGP#ACOE438 CBGP#ACOE2052	*Anuraphis shaposhnikovi* *Anuraphis shaposhnikovi* *Anuraphis shaposhnikovi*	*Opopanax chironium* Not reported Not reported	37,6324N/14,9859E Belpasso (Catania, Sicily) 44,1891N/6,7477E Entraunes (France) 37,9216N/14,957E Randazzo (Catania, Sicily)	21 Apr. 14 24 Jul. 98 30 May 06	KT878811 ACEA035 ACEA882
S12413	*Nearctaphis bakeri* (Cowen)	*Trifolium pratense*	45,0877N/7,6387E Torino (Piemonte)	16 Apr. 12	KT878807
S13562 CBGP#ACOE824 CBGP#ACOE1020	*Nearctaphis bakeri* *Nearctaphis bakeri* *Nearctaphis bakeri*	*Trifolium pratense* Not reported Not reported	41,2367N/13,9319E Sessa Aurunca (Caserta, Campania) 43,7337N/3,5500 Saint-Guillerme-le-Desert (France) 47,9862N/-4,4642E Plouhinec (France)	12 Jun. 13 8 Apr. 00 30 Jul.00	KT878806 ACEA242 ACEA331
S06340 CBGP#ACOE460	*Aphis fabae* Scopoli *Aphis fabae*	*Vicia faba* Not reported	36,9251N/14,7423E Ragusa (Sicily) 44,0105N/3,6058E Levignan (France)	20 Apr. 06 1 Jul. 98	KT878822 ACEA050
S04230	*Roepkea marchali* HRL	*Prunus mahaleb*	43,2235N/13,1518E S. Severino (Macerata, Marche)	20 May 04	KT878812
S14613	*Roepkea marchali*	*Prunus mahaleb*	50,0810N/14,4029E Prague (Czech Rep.)	31 May 14	KT878813
S14623 CBGP#ACOE1674	*Roepkea marchali* *Roepkea marchali*	*Prunus mahaleb* Not reported	50,0871N/14,4172E Prague (Czech Rep.) 43,6833N/3,9262E Teyran (France)	1 Jun. 14 26 Jun. 0	KT878814 ACEA723
S03145 GBMIN10086	*Brachycaudus jacobi* Stroyan *Brachycaudus jacobi*	*Myosotis sylvatica* *Myosotis sylvatica*	38,0505N/15,4343E Itala (Messina, Sicily) 38,0505N/15,4343E Itala (Messina, Sicily)	15 May 03 15 May 03	EU189690 EU196598

Whole aphid specimens for DNA sequencing were stored in 95% ethanol at -20 °C, those used for morphological observations were stored in 70% ethanol and at room temperature.

Total genomic DNA was extracted by macerating entire single individuals using the DNeasy Blood & Tissue kit (Qiagen®, Hilden, Germany) in 50 µl of extraction buffer and stored at -20 °C. To compare the utility of the 5’, barcode region, and the 3’ region of COI we amplified the following regions: for the 5’ end, a 600 bp region using primers LCO1490 and HCO2198 (Folmer et al. 1994), widely used on a variety of organisms as well as aphids (Hebert et al. 2003, Coeur D’acier et al. 2008; Kim et al. 2010; Lee et al. 2014), for the 3’ end, a 648 bp fragment using primers C1-J-2195 and TL2-N-3014 (Simon et al. 1994), found to be informative in several aphid studies (Coeur d’acier et al. 2008; Massimino Cocuzza and Cavalieri 2014). PCR reactions were performed using 8.5 µl of buffer premix 2x F (FailSafe tm PCR Premix Selection Kit –Epicentre Technologies) 1 µl of each primer (10 µM), 0.5 µl Taq polymerase (Life Technologies) and 2 µl DNA template (quantified in 6-18 ng/ µl) in a total volume of 21 µl. The cycle conditions for primer set LCO1490 and HCO2198 was 94 °C for 3 min (initial denaturation), followed by 35 cycles of 94 °C for 30 s (denaturation), 48 °C for 1 min (annealing) and 72 °C for 1 min (extension). Primer set C1-J-2195 and TL2-N-3014 conditions were 96 °C for 5 min (initial denaturation) and 35 cycles of 96 °C for 5 s (denaturation), 45 °C for 1 min (annealing), 72 °C for 1 min (extension). PCR products were run in 1.6% agarose gels stained with Syber Safe DNA gel stain (Life Technologies). PCR products were sequenced at BMR genomics (Padua, Italy) or at the W. M. Keck Center at the University of Illinois (Urbana-Champaign, IL) and run on an ABI PRISM 3730XL DNA analyzer (Life Technologies Corporation, Carlsbad, CA, USA). For each sample 2–8 individuals were sequenced, and one representative sequence for each sample was subsequently chosen. Sequences of *Anuraphis* available in Genbank and or BOLD databases were utilized in the analysis and are identified in Table 1 by their accession number.

The COI sequences were edited manually using BioEdit (Hall 1999) or Sequencher v. 5.0 (GeneCodes Corporation, AnnArbor, MI, USA). Nucleotide sequences were translated using EPoS (Griebel et al. 2008) to check for stop codons (Zhang and Hewitt 1996). Sequence divergences were calculated using the *p*-distance model as suggested by Srivathsan and Meier (2012), and a neighbour-joining (NJ) tree (Saitou and Nei 1987), as implemented in MEGA 6 (Tamura et al. 2011), was used to visualize the distance matrix among taxa and population samples. The Bayesian phylogenetic analysis was conducted using Mr.Bayes v 3.2.1 (Ronquist et al. 2012) implementing the GTR + I model of sequence evolution selected by JModel test 2.1.4 (Posada 2008) based on the Akaike information criterion (AIC). Beginning with random trees, four independent runs with four Markov chains were run for 25,000,000 generations. Bayesian trees were sampled every 1000^th^ generations. All other parameters were set at default. Convergence was assessed using TRACER 1.6 (Rambaut et al. 2014) using a 25% burn in value. Posterior probabilities (pp) and the consensus trees were computed in MrBayes. The Bayesian analysis was run on the CIPRES Science Gateway (Miller et al. 2010). A maximum likelihood analysis was also performed using RAxML v. 8 (Stamatakis 2014) with the GTR +I model; clade support for the maximum likelihood tree was determined in RAxML by bootstrap, based on 1000 pseudoreplicates.

## Results

COI was easily amplified for all specimens analysed using the primers indicated above. No frame shift or premature stop codons were detected.

The five prime end (5’) constituted a 601 base pair (bp) fragment. With total bp frequencies of 75.3% for A/T and 24.7% for G/C. These latter results concur with those found for other aphid species (Shufran et al. 2000; Wang et al. 2011). The 5’ end showed that there were 533 conserved and 125 variable nucleotides with 92 of the latter being parsimony informative. The overall average distance for the 5’ end of the COI gene was 5.8, ranging from 0 (samples within a species) to 11.7 across species.

The three prime end (3’) sequences analysed consisted of 648 bp with frequencies of 74.9% A/T and 25.1% G/C. The 3’ end showed that there were 521 constant and 127 variable sites of which 111 were parsimony informative. The percentage of variable sites was slightly higher for the 3’ (19.6%) than the 5’ end (18.99%).

Considering the 5’ region, the mean genetic distance of *Anuraphis* species from *Nearctaphis bakeri*, *Roepkea marchali*, *Brachycaudus jacobi* and *Aphis fabae* were 6.5%, 6.7%, 8.0% and 9.2%, respectively, whereas slightly higher distance values were observed for most comparisons of the 3’ region (7.5%, 7.9, 8.1 and 8.6%, respectively). The genetic differences recorded in the 5’ barcode region among *Anuraphis* species (Table 2) ranged from 0.2% (between *Anuraphis shaposhnikovi* and *Anurahis catonii*) to 6.7% (between *Anuraphis cachryos* and *Anuraphis pyrilaseri*). When the 3’ region was used, the pairwise distance ranged from 0.8 (*Anuraphis shaposhnikovi* vs *Anurahis catonii*) to 7.4 (*Anuraphis subterranea* vs *Anuraphis pyrilaseri*).

**Table 2.** *p*-distance and nucleotide divergences (expressed as percentage) of *Anuraphis* spp. and species used as outgroup.

		1		2		3		4		5		6		7		8		9	
		5’	3’	5’	3’	5’	3’	5’	3’	5’	3’	5’	3’	5’	3’	5’	3’	5’	3’
**1**	*Anuraphis farfarae*																		
**2**	*Anuraphis pyrilaseri*	1.7	3.2																
**3**	*Anuraphis subterranea*	5.7	7.2	5.8	7.4														
**4**	*Anuraphis shaposhnikovi*	5.3	6.9	5.0	6.6	3.7	4.7												
**5**	*Anurahis catonii*	5.5	6.9	5.2	6.6	3.9	4.8	0.2	0.8										
**6**	*Anuraphis cachryos*	6.6	7.0	6.7	6.6	4.3	5.9	5.6	3.3	5.6	3.8								
**7**	*Nearctaphis bakeri*	6.8	8.3	6.9	7.9	5.6	7.6	6.6	6.9	6.6	7.0	6.7	7.1						
**8**	*Roepkea marchali*	7.3	7.9	6.7	8.2	6.2	8.0	6.7	7.6	6.7	7.7	6.8	8.1	5.5	7.4				
**9**	*Brachycaudus jacobi*	8.5	8.4	8.9	8.5	7.1	8.5	7.8	7.5	7.8	7.6	7.8	8.2	7.5	8.0	6.9	6.8		
**10**	*Aphis fabae*	10.0	9.9	9.1	8.1	9.1	9.1	9.0	7.9	9.0	8.0	8.9	8.4	8.1	8.1	8.5	10.0	10.0	9.5

Our results indicate that there is high genetic homogeneity within *Anuraphis* species, despite differences in geographic origin and host plant. *Anuraphis farfarae* is the only member of the genus that uses Asteraceae, nevertheless its position in *Anuraphis* is well supported (Fig. 2c and 2a). Adaptation to this host plant may be of recent origin and its ecological uniqueness is not reflected at the COI level.

Little to no intraspecific differences were found among the various geographic samples of each *Anuraphis* species (0.3% only for some populations of *Anurahis catonii*, *Anuraphis cachryos* and *Anuraphis pyrilaseri*). Phylogenetic analysis with Neighbour Joining (NJ), Maximum Likelyhood (ML) and Bayesian (MrBayes) using the 5’ and 3’ end of the COI gene showed two discreet clades: one comprising *Anuraphis farfarae* and *Anuraphis pyrilaseri*; the other including *Anuraphis cachryos*, *Anuraphis subterranea*, *Anurahis catonii*, and *Anuraphis shaposhnikovi* respectively (Figs 1, 2).

**Figure 1a.**
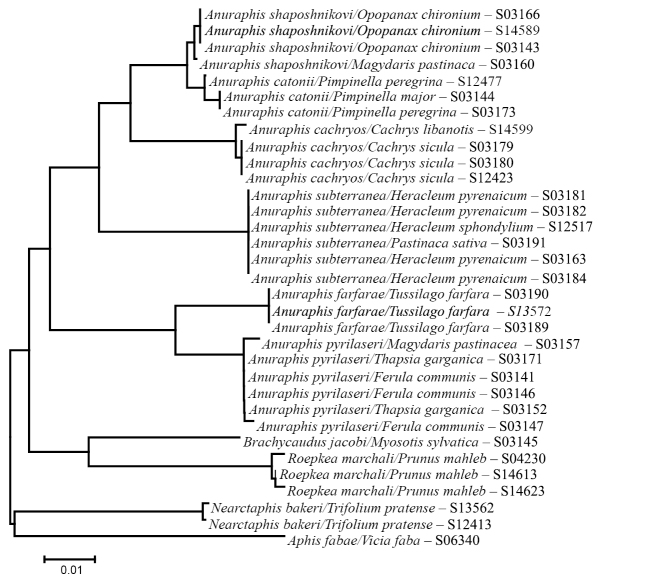
Neighbor-Joining tree showing relationships among selected *Anuraphis* species estimated using 648 bp at the 3’ end of the COI mitochondrial gene. Distance were estimated using the *p*-distance model of sequence evolution.

**Figure 1b.**
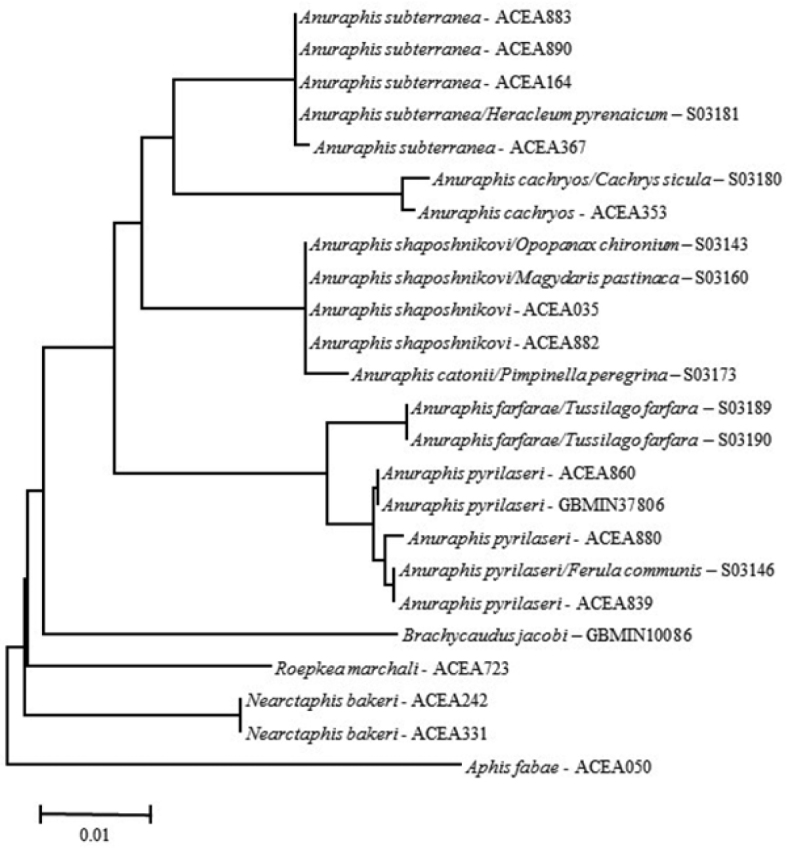
Neighbor-Joining tree showing relationships among selected *Anuraphis* species estimated using 658 bp at the 5’ end of the COI mitochondrial gene. Distance were estimated using the *p*-distance model of sequence evolution.

**Figure 2a.**
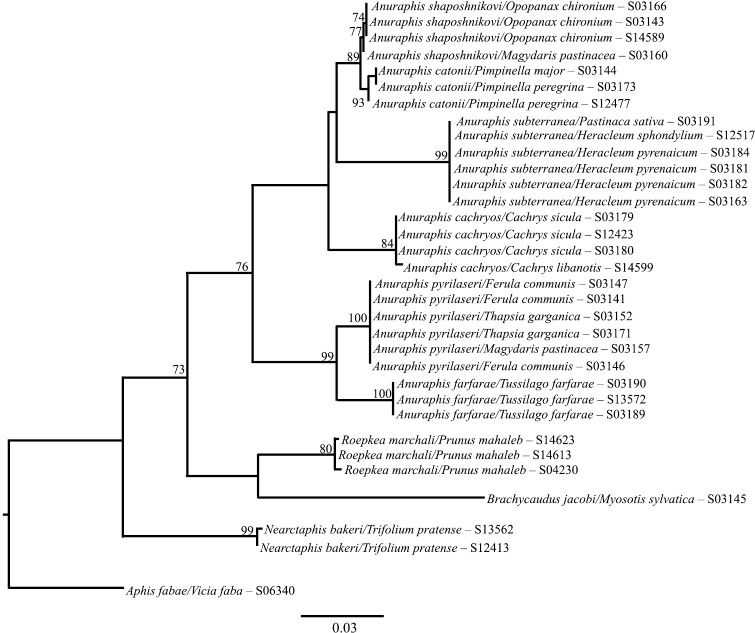
Likelihood tree estimated using 648 bp at the 3’ end of COI for selected *Anuraphis* species.

**Figure 2b.**
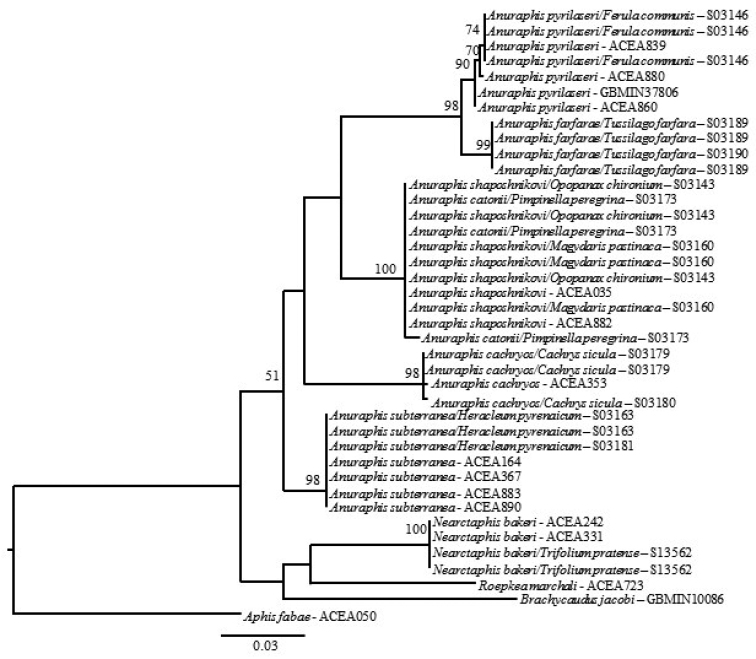
Likelihood tree estimated using 658 bp at the 5’ end of COI for selected *Anuraphis* species.

**Figure 2c.**
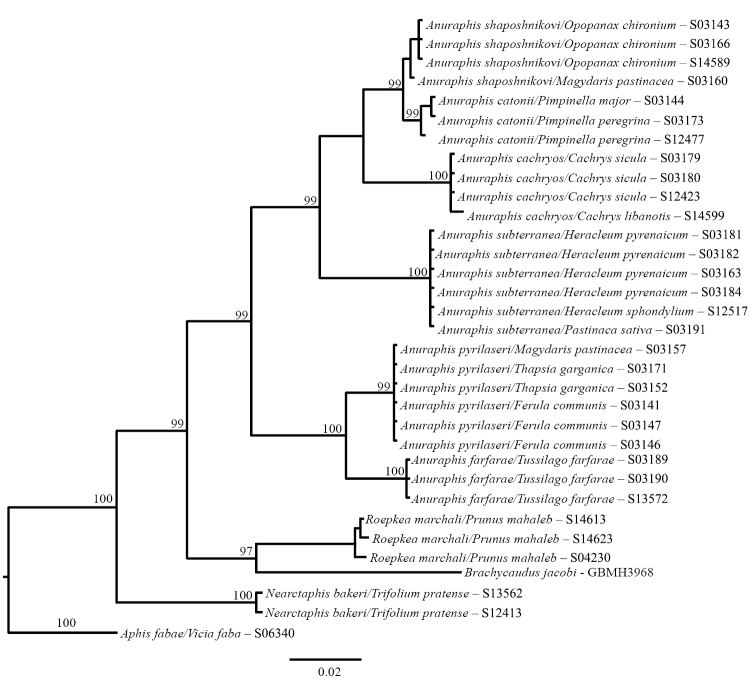
MrBayes tree estimated using 648 bp at the 3’ end of COI for selected *Anuraphis* species.

**Figure 2d.**
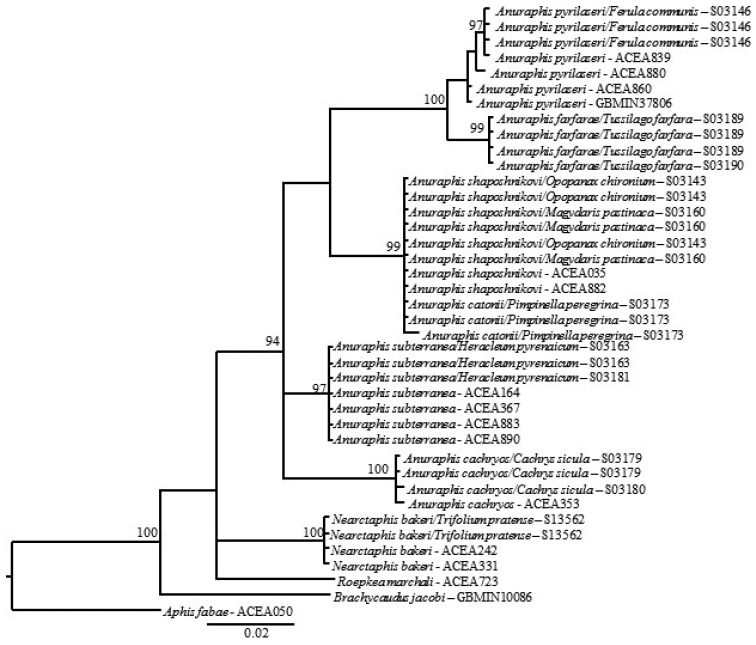
MrBayes tree estimated using 658 bp at the 5’ end of COI for selected *Anuraphis* species.

The clade including *Anuraphis farfarae* and *Anuraphis pyrilaseri* shows a genetic distance between the two species of 3.2% when using the 3’end and 1.7% when using the 5’ end of COI. The various samples of *Anuraphis farfarae* were highly similar, regardless of host plant, locality and COI region examined. Similarly, the populations of *Anuraphis pyrilaseri* showed low genetic variability (0.3%). Differences in body colour, possibly due to host plant effects, as well as differences in dorsal abdominal sclerotisation, do not correlate with the low genetic diversity observed with the COI gene. The various samples of *Anuraphis subterranea* showed no genetic differences, regardless of their geographic origin, host plant or COI region used for the analysis. Genetic difference (3.7% with 3’ and 4.7% with 5’ region) between *Anuraphis subterranea* and *Anuraphis shaposhnikovi* clearly distinguishes the two species, despite the small morphological differences observed (length of ultimate rostral segment and number and distribution of abdominal spinal tubercles). *Anuraphis shaposhnikovi* and *Anurahis catonii* showed the lowest genetic divergence (<1%) regardless of the COI region considered. However, while with 5’ COI barcode showed a pairwise distance of 0.2%, the 3’ region showed a difference of 0.8%.

**Figure 3.**
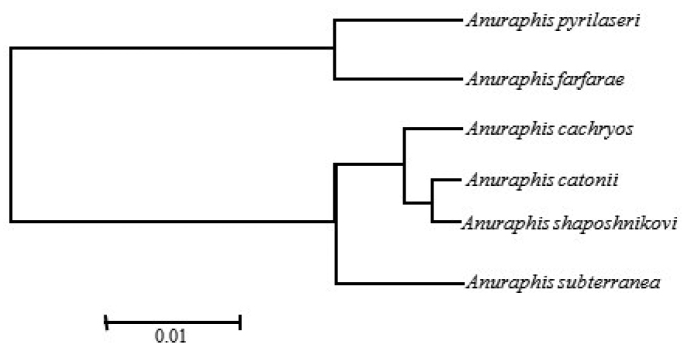
Dendrogram of cluster-species results based on Mahalanobis’ generalized distances in apterae for *Anuraphis* spp. (20 individual for each species) based on 16 morphometric characters (from Barbagallo and Cocuzza 2003).

A result similar to the one based on COI was found using a multivariate discriminant analysis with 16 morphometric characters (Barbagallo and Cocuzza 2003) and graphically as Mahalanobis’ generalized distance (Fig. 3). The dendrogram indicates a distinction of *Anuraphis subterranea* and *Anuraphis shaposhnikovi*, and the similarity between the latter species and *Anurahis catonii*.

## Discussion

The molecular analysis based on the 3’ and 5’ COI gene regions indicates that the genus *Anuraphis* is a homogeneous taxonomic group. However, COI also provides information to distinguish the taxa at the species level as evidenced by the level of support, 89% bootstrap or more, on the likelihood tree (Fig. 2a). Thus, the analysis using COI confirms the species delimitation concepts previously reported using a multivariate analysis of morphological features (Barbagallo and Cocuzza 2003). The division of *Anuraphis* species in two groups (one clade consisting of *Anuraphis farfarae* and *Anuraphis pyrilaseri*, a second clade including *Anuraphis subterranea*, *Anuraphis cachryos*, *Anuraphis shaposhnikovi* and *Anurahis catonii*) is easily observable by comparing the phylogenetic trees and Mahalanobis’ generalized distance. The COI-based molecular analysis permitted a better discrimination of *Anuraphis shaposhnikovi* and *Anuraphis subterranea* than the multivariate analysis based on morphometric features. It is useful that the COI gene can also differentiate *Anuraphis subterranea* and *Anurahis catonii*, because the taxonomic status of the latter species has been questioned. Hille Ris Lambers (1935), regarded *Anurahis catonii* as a subspecies of *Anuraphis subterranea*. The only morphological difference between *Anuraphis subterranea* and *Anurahis catonii* noted by Stroyan (1950) was in the number of secondary rhinaria on the antennae of alatae, more numerous in the former species. However, Blackman (2010) has reported other morphological differences between these two species, both in apterae and alatae. Biologically, it has been shown that when transferred to *Pastinaca sativa*, the nymphs of *Anurahis catonii* can reach adulthood (Stroyan 1959); conversely, Shaposhnikov (1951) observed that nymphs of *Anurahis catonii* transferred from pear survive on *Pimpinella* sp. but not on *Pastinaca sativa*. A further intricacy was the recovery by Kolesova (1972) of a sample of *Anurahis catonii* on *Pastinaca sativa*, although this could be a case of misidentification.

Barbagallo and Cocuzza (2003) reported that *Anuraphis shaposhnikovi*, collected on *Magydaris pastinacea* has slight morphological differences from those developing on *Opopanax chironium*, (*i.e*., the length of the last rostral segment and the number of abdominal spinal tubercles). The putatively fixed nature of the morphological differences is confirmed by the COI analysis and can be the result of intraspecific variability and possibly geographic isolation, since *Magydaris pastinacea* occurs in very restricted areas of Sicily and Sardinia. Another interesting observation is the low genetic divergence observed between *Anurahis catonii* and *Anuraphis shaposhnikovi*, a similarity already evidenced in the morphological analysis (Barbagallo and Cocuzza 2003). These species may have diverged recently from a common ancestor as a result of differences in the habitats of their respective host plants. The genus *Pimpinella* is typical of herb-rich areas and wooded pastures, whereas *Opopanax chironium* prefers uncultivated dry land with a Mediterranean climate (Pignatti 1982). The phenomenon of host-races as a first step leading to speciation has been repeatedly observed in phytophagous insects (Drès and Mallet 2001) and is common in aphids (Sunnucks et al. 1997; Margaritopoulos et al. 2007), especially in populations that have partially or totally lost the sexual generation in favour of continuous parthenogenetic reproduction. Host-plant use may represent a food resource niche that favours the speciation process of species in sympatry (Peccoud et al. 2010). Moreover, low genetic diversity at the COI level is typical of taxa with recent ecological divergence (Jimbo et al. 2011) and can explain the low genetic divergence (<1%) reported in some aphid groups (Foottit et al. 2008; Lee et al. 2011; Massimino Cocuzza and Cavalieri 2014). Lee et al. (2014) found that the COI barcode region was not helpful in the identification of 7% of the aphid species they examined. This lack of resolution could be resolved by the development of additional molecular markers with higher diversity, leading to greater accuracy in species identification (Lozier et al. 2009; Sano and Akimoto 2012; Chen et al. 2013; Lee et al. 2014). In the case of *Anurahis catonii* and *Anuraphis shaposhnikovi* the genetic difference, albeit low, was consistently observed in all samples analysed.

We observed a difference in genetic distances when using the 5’ barcode or the 3’ regions of COI. Most “barcode” studies on aphids are carried out using the 5’ region of COI that has produced some ambiguous results (Foottit et al. 2008; Žuroková et al. 2010; Lee et al. 2011). This study demonstrates that in *Anuraphis* the 3’ COI region has a higher capacity of discrimination. In the case of *Anurahis catonii* and *Anuraphis shaposhnikovi* the difference recorded with the 3’ (0.8%) and 5’ regions (0.2%) is crucial, especially when considering that a distance of 0.5% in aphids is usually considered as the “borderline” between species (Massimino Cocuzza and Cavalieri 2014; Rakauskas et al. 2014). However, low genetic difference in species that are morphologically different is not an unknown phenomenon in aphids. For example, despite *Aphis hederae* Kaltenbach, 1843 and *Aphis newtoni* Theobald, 1927 having well-defined morphological and biological differences, they have a low interspecific divergence (0.17%) in the 5’ COI region (Lee et al. 2014).

The genetic results observed here in *Anuraphis* spp. closely mirror previous morphometric findings. The lack of appreciable differences in morphological characters is a phenomenon well known in various groups of aphids (Stroyan 1984; Foottit 1997; Wang et al. 2011) and this peculiarity can easily lead to the misidentification of species (Coeur d’acier et al. 2007). Because of this difficulty, there is a need for methods of investigation that can be used in conjunction with classic morphometric analysis. Confirming the finding of previous studies on aphids (Foottit et al. 2008; 2009c), the present study indicates that the COI gene may significantly aid in the correct identification of aphid species, especially in cases where morphological characters are insufficient to clarify taxonomic status. Morphometrics and the COI gene can be used in parallel to improve the discrimination of aphid species. However, an identification-integrated system that links molecular data, morphological features, life cycle, host plant, photos (in vivo and on slides) and a bibliography for each aphid species would further facilitate and improve the accuracy of aphid species determination.
